# Mutagenesis of the L, M, and N Subunits of Complex I from *Escherichia coli* Indicates a Common Role in Function

**DOI:** 10.1371/journal.pone.0017420

**Published:** 2011-02-28

**Authors:** Jose Michel, Jessica DeLeon-Rangel, Shaotong Zhu, Kalie Van Ree, Steven B. Vik

**Affiliations:** Department of Biological Sciences, Southern Methodist University, Dallas, Texas, United States of America; University of Cambridge, United Kingdom

## Abstract

**Background:**

The membrane arm of Complex I (NADH:ubiquinone oxidoreductase) contains three large, and closely related subunits, which are called L, M, and N in *E. coli*. These subunits are homologous to components of multi-subunit Na^+^/H^+^ antiporters, and so are implicated in proton translocation.

**Methodology/Principal Findings:**

Nineteen site-specific mutations were constructed at two corresponding positions in each of the three subunits. Two positions were selected in each subunit: L_K169, M_K173, N_K158 and L_Q236, M_H241, N_H224. Membrane vesicles were prepared from all of the resulting mutant strains, and were assayed for deamino-NADH oxidase activity, proton translocation, ferricyanide reductase activity, and sensitivity to capsaicin. Corresponding mutations in the three subunits were found to have very similar effects on all activities measured. In addition, the effect of adding exogenous decylubiquinone on these activities was tested. 50 µM decylubiquinone stimulated both deamino-NADH oxidase activity and proton translocation by wild type membrane vesicles, but was inhibitory towards the same activities by membrane vesicles bearing the lysine substitution at the L236/M241/N224 positions.

**Conclusions/Significance:**

The results show a close correlation with reduced activity among the corresponding mutations, and provide evidence that the L, M, and N subunits have a common role in Complex I.

## Introduction

Complex I (NADH:ubiquinone oxidoreductase) is the initial electron acceptor of the mitochondrial respiratory chain, and it is a key member of the electron transport chains of many bacteria (for a review see [1]). It is a membrane-bound, multi-subunit enzyme, and as found in mitochondria it is composed of up to 45 distinct protein chains [2]. It contains one flavin mononucleotide and up to 9 Fe-S centers. Functionally, the core enzyme has three discrete roles: (1) It regenerates NAD^+^ from NADH, thereby allowing the citric acid cycle to continue. (2) It reduces ubiquinone so that the electron transport chain can function. (3) It conserves energy by translocation of protons, or possibly other cations, across the membrane. The mitochondrial enzymes are likely to be involved in several other activities.

Complex I from various species has been shown to have an “L” shape, consisting of a membrane arm and a peripheral arm [3], [4]. Physically and conceptually, they can be separated: a peripheral arm that contains all of the prosthetic groups involved in electron transport, and a membrane arm that contains all of the integral membrane proteins. In the bacterium *E. coli*, the peripheral arm comprises 6 subunits called B, CD, E, F, G and I, where CD can be considered the result of a fusion of 2 genes that are distinct in many other organisms. The membrane arm comprises 7 subunits called A, H, J, K, L, M, and N, which are homologous to the mitochondrially coded subunits of Complex I in mammals. In *E. coli*, all 13 genes constitute the *nuo* operon [5].

The properties of the Fe-S centers have been studied primarily by EPR spectroscopy [6], [7], [8], [9], [10]. They were first visualized by crystallography in 2006, following the solution of the structure of a bacterial peripheral arm from *Thermus thermophilus* [11]. FMN is bound near one end of the complex, adjacent to the NADH binding site. A series of seven Fe-S centers are found along a nearly linear path. The center most distal from the FMN is the N2 site, which is found in subunit B, and adjacent to subunit CD. An eighth center is on the opposite side of the FMN, the N1a center. A ninth center was found in subunit G, at some distance from any other Fe-S, and it is thought not to be part of the electron transport pathway [12]. This center is also present in the *E. coli* enzyme, but not in mitochondrial ones. The N2 Fe-S center has the highest redox midpoint potential of the prosthetic groups in Complex I, and is thought to directly reduce ubiquinone [10], [13]. Mutagenic analysis has supported this view [14], [15].

The membrane arm has been visualized at low resolution by cryoelectron microscopy [16], and more recently at higher resolution by X-ray diffraction at 3.9 Å [17] and 6.3 Å [18]. Subunit H is thought to provide the greatest interaction with the B and CD subunits of the peripheral arm. Analysis of sub-complexes has indicated that subunit J is associated with H, and that subunits L, followed by M, are at the distal end of the membrane arm [19]. Based on this information, and the size of the L, M, and N subunits, it is likely that the membrane arm that protrudes from the junction consists primarily of subunits N, M and L, in that order. In *E. coli*, the M and N subunits are very similar in size, 485 and 509 amino acids, while subunit L has 613 amino acids. The additional, nonhomologous, residues of L are clearly located at the C-terminus. The recent crystal structure suggests that each of the three subunits contains fourteen similarly oriented transmembrane helices [17]. The C-terminal region of subunit L appears to contain two additional transmembrane helices connected by a lateral helical segment that extends parallel to the membrane surface, at the cytoplasmic side. One transmembrane helix is found at the distal end of the membrane arm, while the other is located near the N subunit at the junction of the two arms. The overall membrane topology of subunits M (*E. coli*) and subunit L (*Rhodobacter capsulatus*) had been addressed experimentally earlier [20], [21], but some questions remained. An analysis of the membrane topology of subunit N from *E. coli* was recently reported [22] that supports a 14 transmembrane helix model.

The mechanism of proton translocation by Complex I is not clear, but surely must involve ubiquinone/ubiquinol and the membrane subunits. The L, M, and N subunits are primary candidates for this function because they are homologous to two proteins, MrpA and MrpD, in a multi-subunit Na^+^/H^+^ antiporter complex found in bacteria [23], [24]. Subunit K, the smallest of the *E. coli* subunits, is homologous to MrpC, of the same antiporter family [25]. Evidence indicates that there are two binding sites in Complex I for ubisemiquinone [8], [13], [26]. One site, Q_Nf_, is within 12 Å of the N2 Fe-S center, and the other, Q_Ns_, is at a rather greater distance, presumably within the membrane arm. One possibility that has been considered is that the ubiquinone functions as a mobile proton carrier, similar to the Q cycle in Complex III [27], [28]. The difference with Complex I is that there are no known prosthetic groups in the membrane subunits to couple proton and electron transfer. While there is no direct evidence for quinone binding sites in the membrane subunits of Complex I, indirect evidence has suggested that the L, M, N subunits might interact with quinones. Several studies have found that photo-affinity cross-linkers that are derivatives of ubiquinone, or of inhibitors of Complex I, can form covalent bonds to subunits L [29], M [30], or N [31].

Complementary to these studies was a mutagenic analysis of subunit N in *E. coli* [32]. About twenty site-specific mutations were constructed among conserved residues. Among these, several were particularly interesting, in that deamino-NADH oxidase activity in membrane vesicles from several mutants was not stimulated by addition of decylubiquinone. In particular, mutations at residues E154, K158, H224, and Y300 showed no stimulation, or even inhibition. The current work was motivated by the questions: Do the L, M, and N subunits all function in a similar way, and do they respond similarly to decylubiquinone. To answer these questions, a series of mutations was constructed in all three subunits at positions corresponding to N_K158 and N_H224.

## Results

**Figure 1:**
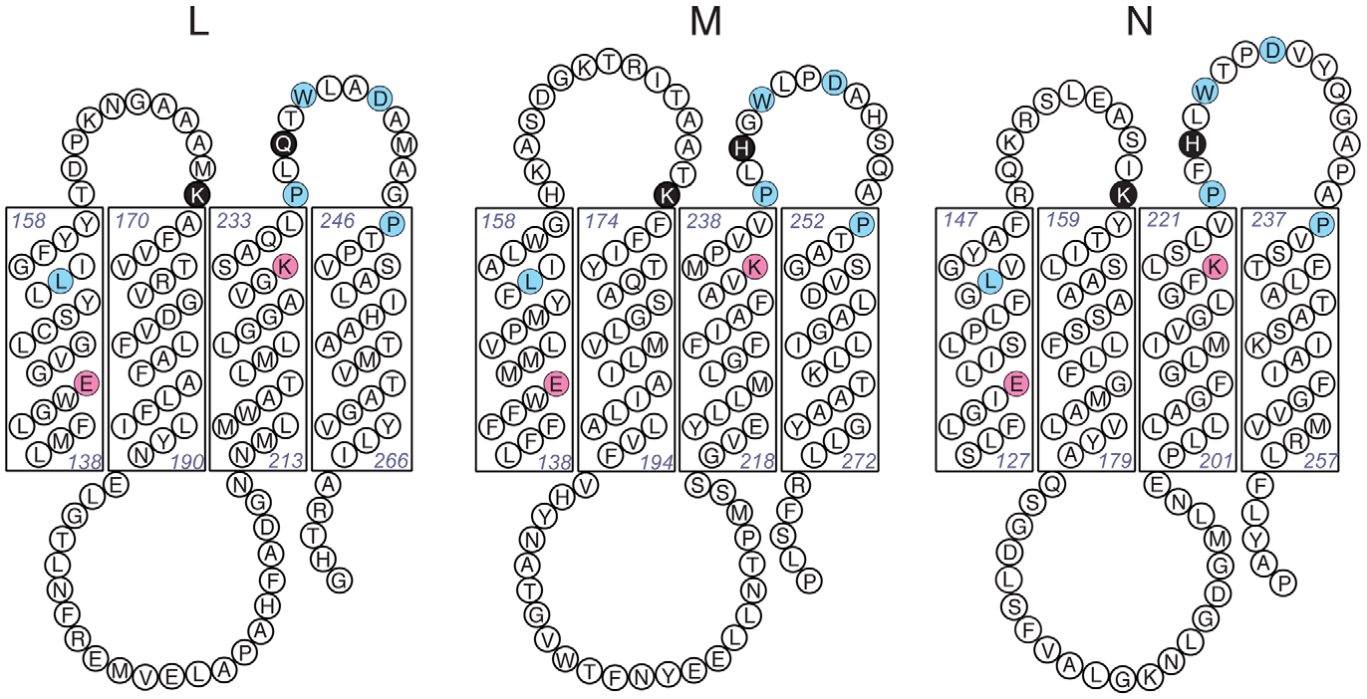
Comparison of subunits L, M and N in the central regions of the proteins. Four corresponding transmembrane helices of each subunit are shown, with the cytoplasm above and the periplasm below. The sites of mutations generated in this study are the two residues shaded black in each protein: L_K169, L_Q236, M_K173, M_H241, N_K158, N_H224. Two residues, shaded red, are highly conserved among Complex I homologues: one glutamic acid (L_E144, M_E144, and N_E133) and one lysine (L_K229, M_K234, and N_K217). Other residues that are conserved among all three subunits in *E. coli* are indicated by circles shaded in blue.

**Figure 2:**
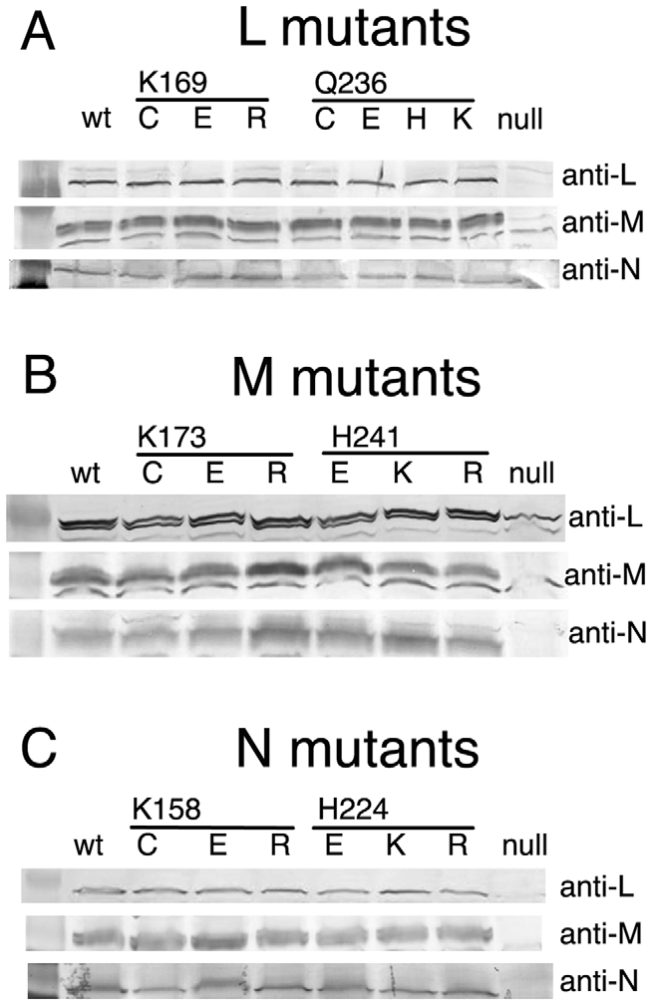
Immunoblots of membrane preparations from all nineteen mutants. Three independent blots were performed with each mutant, using antibodies against subunits L, M, or for detection of N, anti-HA antibodies. For comparison, the null strain, BA14, and the wild type strain, BA14/pBA400, were included in each blot. Each lane contained 40 µg of protein. (A) Seven L mutants. (B) Six M mutants. (C) Six N mutants.

In a previous study [32], mutations at numerous positions of subunit N of the *E. coli* Complex I resulted in the loss of enzyme activity, but several mutations at E154, K158, H224 and Y300 also caused reduced ability to utilize exogenous decylubiquinone in NADH oxidase activity. In this study, the residues homologous to K158 (position 1) and H224 (position 2) were selected in subunit L (K169, Q236) and in subunit M (K173, H241), as shown in Figure 1. These residues are highly conserved among bacterial L, M and N subunits. They were selected in part because of their proximity to two residues that are highly conserved in Complex I subunits from nearly all species, and which might be involved in proton translocation: E (L144, M144, N133) and K (L229, M234, N217) [32], [33], [34], [35], [36]. At least three mutations were constructed at each of the six sites, resulting in 19 in total. Each mutation was transferred to the *nuo* operon expression vector, pBA400, and the resulting plasmids were used individually to transform the *nuo* deletion strain BA14. All of the mutants, which are listed in Table 1, could grow on minimal medium plates with acetate as the sole carbon source, indicating a functional, or partially functional, Complex I. Each of the mutant strains was further analyzed by immunoblotting of membrane vesicle preparations for all three subunits L, M, and N. Peptide-based antibodies were used to detect subunits L and M, and subunit N was detected via an engineered HA epitope tag at its C-terminus. The results, shown in Figure 2, indicate that the levels of all of the subunits are very similar to the strain with the wild type plasmid.

**Table 1:** Activity measurements of nineteen mutants of the L, M, and N subunits.

Mutation	Deamino-NADH oxidase activity (nmoles/mg protein/min) a	% Deamino-NADH oxidase activity b	Ferricyanide reductase activity (nmoles/mg protein/min) a	% Ferricyanide reductase activity b	Capsaicin sensitivity (IC_50_) c
wild type	231±16 (18) d	100	1690±120 d	100	<25 µM
L_K169C	151±13 (4)	65	1439±300	87	ND e
L_K169E	155±18 (4)	67	1989±200	117	<50 µM
L_K169R	217±16 (3)	94	2001±320	118	ND
L_Q236C	199±15 (5)	86	1790±100	106	<25 µM
L_Q236E	193±13 (4)	84	1990±130	117	<50 µM
L_Q236H	198±8 (6)	86	1830±150	108	<50 µM
L_Q236K	132±9 (8)	57	1680±260	99	ND
M_K173C	162±14 (3)	70	1320±40	78	<25 µM
M_K173E	116±9 (5)	50	1720±120	102	<25 µM
M_K173R	210±6 (5)	91	1580±200	94	<25 µM
M_H241E	163±2 (3)	71	1340±30	79	ND
M_H241K	93±16 (3)	40	1590±30	94	ND
M_H241R	107±14 (4)	46	1360±80	81	<25 µM
N_K158C	117±7 (7)	51	1460±80	86	<25 µM
N_K158E	109±9 (4)	47	1200±180	71	<25 µM
N_K158R	161±8 (5)	70	1610±160	95	<25 µM
N_H224E	155±14 (4)	67	1690±140	100	<25 µM
N_H224K	86±12 (6)	37	1610±50	95	<50 µM
N_H224R	73±3 (3)	32	1170±70	69	ND
BA14	10±1 (17)	4	520±90	30	

^*a*^Activity was measured in membrane preparations as described in “Materials and Methods”.

^*b*^Activity is expressed as a percentage of the wild type value.

^*c*^An upper limit is estimated for the IC_50_ values based on two sets of inhibition data.

^*d*^The means, standard deviations and (number of measurements) are shown. For ferricyanide reductase, 2–3 measurements were made.

^*e*^ND, not determined.

Enzyme activity of each mutant was measured in preparations of membrane vesicles, using deamino-NADH as the substrate for NADH oxidase activity. Deamino-NADH is the hypoxanthine variant of NADH, which cannot be utilized by the alternative NADH dehdyrogenase (NDH-2) that is also found in the membranes of *E. coli* [37]. For each mutant, a second deamino-NADH assay was performed, using potassium ferricyanide as the terminal electron acceptor. The results of this assay provide an indication of the quantity of the peripheral arm of Complex I in preparations of membrane vesicles, and so is complementary to immunoblots of the membrane subunits. The results of these enzyme assays are shown in Table 1. Also shown are the results of testing sensitivity to capsaicin for most of the mutants. The ferricyanide reductase activities of the mutants were all between about 70 and 120% of the wild type rate. Similarly, the sensitivity of deamino-NADH oxidase activity to capsaicin of the thirteen mutants tested were all similar to the wild type.

**Figure 3:**
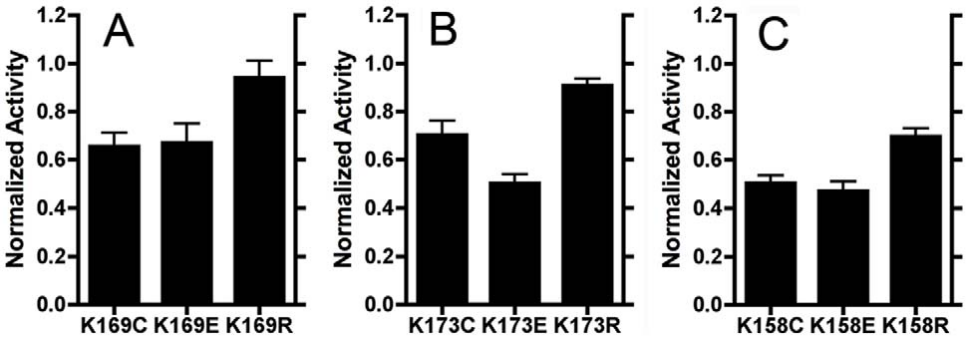
Comparison of mutations at the first site: L_K169, M_K173, and N_K158. Membrane vesicles were prepared and assayed for deamino-NADH oxidase activity. Each assay was initiated with 250 µM deamino-NADH, and included 1 µM FCCP and about 150 µg/ml membrane protein. The rates are plotted relative to the wild type rate, which was typically about 230 nmoles/min/mg protein. The rates shown are the means of 3–7 measurements ± standard deviation. (***A***) L subunit mutations K169C, E, R. (***B***) M subunit mutations K173C, E, R. (***C***) N subunit mutations K158C, E, R.

**Figure 4:**
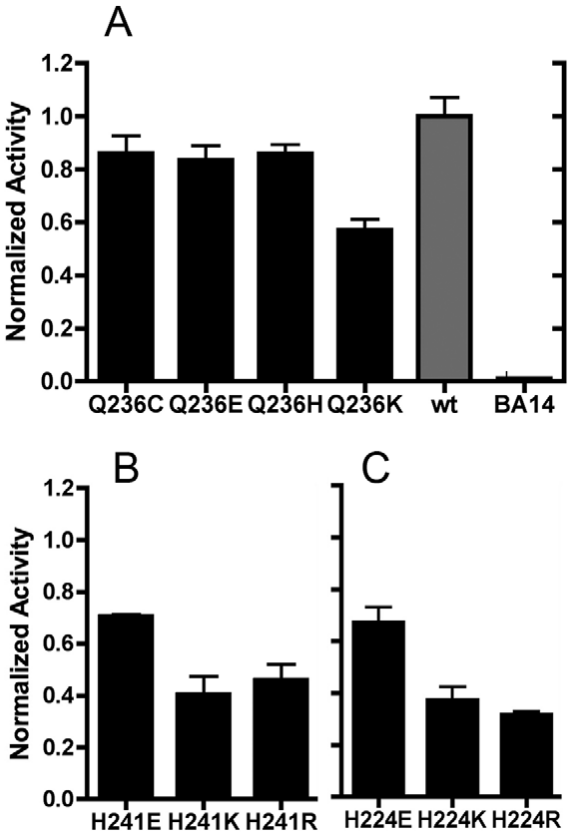
Comparison of mutations at the second site: L_Q236, M_H241, and N_H224. Membrane vesicles were prepared and assayed for deamino-NADH oxidase activity. Each assay was initiated with 250 µM deamino-NADH, and included 1 µM FCCP and about 150 µg/ml membrane protein. The rates are plotted relative to the wild type rate, which was typically about 230 nmoles/min/mg protein. The rates shown are the means of 3–7 measurements ± standard deviation. (***A***) L subunit mutations Q236C, E, H, K. Also shown are rates for the wild type and the null strains. (***B***) M subunit mutations H241E, K, R. (***C***) N subunit mutations H224E, K, R.

The rates of deamino-NADH oxidase activity among the nineteen mutants ranged from 32 to 94% of the wild type rate. The trends can be better observed in Figures 3 and 4. In Figure 3, the rates of the L_K169, M_K173, and N_K158 mutants are expressed as a percentage of the wild type rate. In each subunit, the K→R substitution has the highest rate, while the K→C and E substitutions are lower, and more similar, in each case. Consistent trends can also be observed among the substitutions for L_Q236, M_H241, and N_H224, shown in Figure 4. Results for the wild type strain, and for the null strain, BA14, are also shown in this figure. Substitutions of H→E had the least effect in each subunit. Note that in subunit L this residue is Q, but that substitutions to H and to C also had little effect on the activity. Substitutions to K or to R had more significant effects on activity in all three subunits.

**Figure 5:**
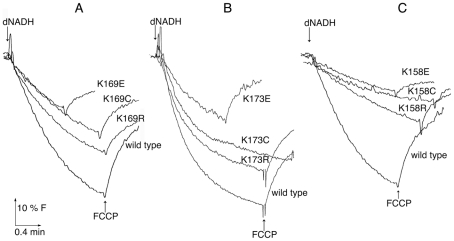
Comparison of proton translocation rates with mutations at the first site: L_K169, M_K173, N_K158. The reactions were initiated with deamino-NADH (dNADH) to 250 µM final concentration. The fluorescence of ACMA (1 µM) was followed for several minutes. The uncoupler FCCP was added (1 µM) to collapse the generated proton gradient. In each panel the wild type strain is shown for comparison. The traces shown are representative of 2–3 experiments. (***A***) L subunit mutations K169C, E, R. (***B***) M subunit mutations K173C, E, R. (***C***) N subunit mutations K158C, E, R.

Proton translocation assays were also carried out in preparations of membrane vesicles. The rates of deamino-NADH driven proton translocation were assessed by the quenching of fluorescence of the acridine dye ACMA (9-amino-6-chloro-2-methoxyacridine). For each of the 19 mutants the rate of proton translocation closely paralleled the rate of deamino-NADH oxidase. The rates for all three mutations at residues L_K169, M_K173, and N_K158, are shown in Figure 5, panels A, B, and C, respectively. Each assay is initiated by the addition of deamino-NADH, and completed with the addition of FCCP (carbonyl cyanide *p*-(trifluoromethoxy) phenylhydrazone), which collapses any proton gradient that has formed. In each panel, the rate of the wild type is rather more than that of the highest mutant, which is the K→R substitution. In each case, the other two substitutions, C and E, have lower rates than R. For comparison with deamino-NADH oxidase rates, see Figure 3.

The effect of decylubiquinone on Complex I activities was examined. The addition of decylubiquinone will increase the wild type rate of NADH oxidation because the endogenous ubiquinone is limiting, and decylubiquinone can be reduced at the normal ubiquinone site [38]. In previous work it was shown that 100 µM decylubiquinone inhibited deamino-NADH oxidase by two mutants, N_K158C and N_H224K, while the wild type was stimulated [32]. This inhibition suggested that quinones might interact with subunit N. In this work similar effects were found with mutations at the L_Q236, M_H241 and N_H224 positions using 50 µM decylubiquinone. In Table 2, the results of deamino-NADH oxidase assays in the presence of 50 µM decylubiquinone are shown. While the wild type is stimulated about 10%, each of the three mutants is inhibited about 10%.

**Table 2:** The effect of exogenous decylubiquinone on deamino-NADH oxidase activity.

	deamino-NADH oxidase activity a
*wild type*	111±5.5% b
L_Q236K	87±3.5%
M_H241K	87±4.1%
N_H224K	88±1.2%

^*a*^Activity was measured in membrane preparations as described in “Materials and Methods”.

^*b*^Activities are expressed relative to the rates measured in the absence of decylubiquinone. The means and standard errors from 3–4 measurements are shown. Typical values of the rates can be found in Table 1.

**Figure 6:**
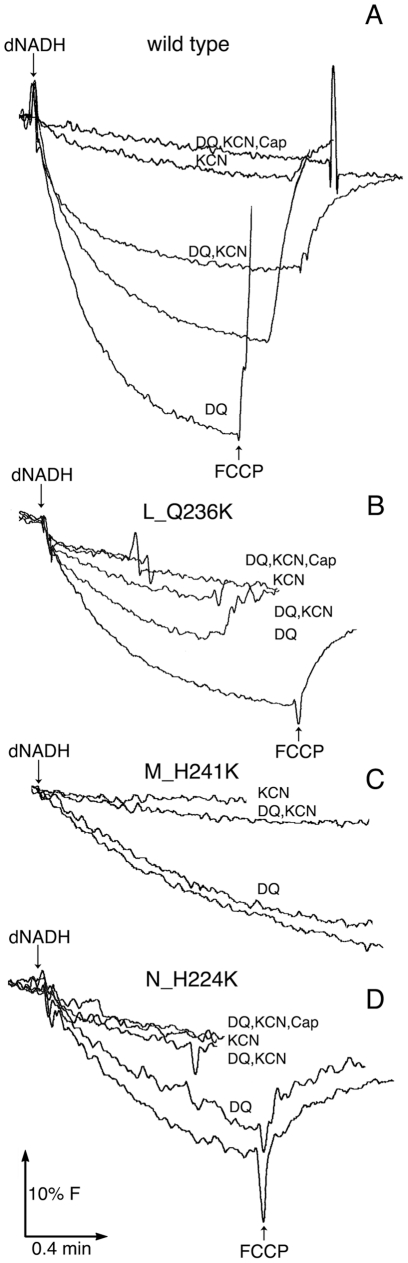
Comparison of the ability to utilize decylubiquinone for proton translocation with mutations at the second site: L_Q236, M_H241, and N_H224. The reactions were initiated by addition of deamino-NADH (dNADH) to 250 µM final concentration. Membrane preparations (150 µM/ml protein) were pre-incubated with 10 mM cyanide (KCN) or 100 µM decylubiquinone (DQ) as indicated in the figure. KCN prevents recycling of the quinones by inhibiting the quinol oxidases. The addition of FCCP (1 µM) collapses the proton gradients, while pre-incubation with capsaicin (Cap) to 300 µM final concentration prevents proton translocation (not shown for all traces). The traces shown are representative of 2–3 experiments. (*A*) wild type. (*B*) L_Q236K. (*C*) M_H241K. (*D*) N_H224K.

A fluorescence quenching assay was developed to test the ability of the Complex I mutants to utilize decylubiquinone for proton translocation. When decylubiquinone is added to membrane vesicles it supplements the endogenous ubiquinones, and in principle, it can be utilized by both Complex I and by the quinol oxidases. If KCN is added, the quinol oxidases become inhibited, and Complex I will continue to function only if the supply of quinones is high enough to support multiple turnovers of the enzyme. In this situation one can test whether an exogenous quinone can be utilized by Complex I. As shown in Figure 6A, proton translocation driven by deamino-NADH in wild type membrane vesicles can be stimulated by the addition of 50 µM decylubiquinone. In the presence of 10 mM KCN, the proton translocation is nearly completely inhibited. However, if decylubiquinone is added to the KCN-inhibited membrane vesicles, the proton translocation rate is nearly the same as the original wild type rate. This indicates that Complex I can utilize decylubiquinone for proton translocation. In all cases tested, the fluorescence quenching can be abolished by the addition of capsaicin, indicating that it is dependent upon the function of Complex I.

Proton translocation by three mutants, L_Q236K, M_H241K, and N_H224K, is shown in Figure 6, panels B, C and D, respectively. In each case, decylubiquinone does not stimulate the rate of proton translocation driven by deamino-NADH. In the presence of KCN, the addition of decylubiquinone provides little or no fluorescence quenching, indicating the inability of those membrane vesicles to utilize that quinone for proton translocation. One possible issue is that the mutant membranes could be particularly permeable to protons, and therefore cannot maintain a significant proton gradient. This possibility was eliminated by control experiments in which proton translocation was measured using regular NADH, which is used by the alternative NADH dehydrogenase (ND-2). All 3 mutants showed the same rapid quenching of fluorescence as did the wild type (results not shown). In a second set of control experiments, the wild type membranes were inhibited by 100 µM capsaicin, so that the rate of proton translocation mimicked that of the 3 mutants. Under these conditions, the ability of decylubiquinone to support proton translocation in the presence of KCN and deamino-NADH was greatly diminished (results not shown). This indicates that low rates of proton translocation may compromise the ability to detect utilization of decylubiquinone in preparations of membrane vesicles.

## Discussion

The similarity of the three largest membrane-bound subunits of Complex I is an intriguing aspect of its function. Recent crystallographic analysis [17] indicated that there is an almost identical three dimensional mapping of 14 transmembrane helices of the L, M and N subunits from *E. coli*. Nevertheless, only about 10% of the residues are conserved among the three subunits in the *E. coli* enzyme, while about 20% of the residues are conserved in each pair wise alignment. Furthermore, the crystallographic analysis [17] confirmed that the subunits are arranged sequentially, from the peripheral arm and Fe-S centers, and L is most distal. The most interesting finding is related to the structure of subunit L, which has two additional transmembrane helices in the C-terminal region that are not found in subunits M or N. These two helices were shown to be connected by a long, nearly continuous alpha-helix that runs along the cytoplasmic surface of the membrane, and in contact with subunits M and N. However, the resolution of the recent crystal structures is not sufficient to identify side chains or transmembrane helix connectivity, and so it is not currently possible to locate any of the mutated amino acids in these structural models.

In this study, two questions were addressed. First, do the three subunits each function in a similar way, and second do mutations in the M and L subunits cause a response to decylubiquinone that is similar to that previously seen in subunit N? In previous studies, individual subunits have been subjected to mutagenesis, such as nuoN [32], nuoM [33], [34], and recently nuoL [35]. The earlier studies have included mutagenesis of the two residues highly conserved among all species: a lysine and a glutamic acid. Mutations at the conserved lysine residue, N_K217, M_K234, or L_K229 (see Figure 1), resulted in loss of function, suggesting a similar mechanism among the three subunits. In the case of the conserved glutamic acid, N_E133, M_E144, and L_E144, mutation to A or Q in subunits M or L caused total loss of activity [33], [35], but mutation to A in subunit N had only modest effects [32]. This highlights one difference in function between the subunits.

The results here support the hypothesis that all three subunits, L, M and N, have a common role in function, even though symmetry considerations require that they also have distinct features. The effects of the mutations are well-correlated among the three subunits. For example, the mutation of the lysines (L_K169, M_K173, and N-K158) to arginine had the least effect on activity (Figures 3 and 5), while mutation to glutamic acid or cysteine had more significant effects. The role of this lysine appears consistent with the positive-inside rule [39], since it is found at the cytoplasmic surface of the membrane. At the other position (L_Q236, M_H241, and N_H224), mutation to a positively charged residue, K or R, had the greatest deleterious effect on activity, while mutation to glutamic acid had little effect (Figure 4). Alanine can also be accommodated without much loss of activity at this position in subunit N [32] or subunit M [33]. Similar results were obtained in a study of the MrpA protein from the Na^+^/H^+^ antiporter form *Bacillus pseudofirmus* OF4 [40], which contains a very similar sequence to that of the three *nuo* proteins. In that protein, H230A had little effect on function, while H230K had only half of the normal transport activity, and an increased K_m_ for Na^+^ ions.

The effect of decylubiquinone on NADH oxidase activity (Table 2) suggests that this water soluble quinone is inhibitory towards the mutants tested, although the observed effects are smaller than those previously reported [32]. There is still no evidence to indicate whether this is the results of a direct interaction between decylubiquinone and L, M, or N subunits. The inhibitory behavior of decylubiquinone is consistent with its inability to be utilized for proton translocation by the same mutants (Figure 6). However, control experiments indicated that low rates of enzyme activity could contribute significantly to that outcome. Therefore, the primary conclusion is that corresponding mutations in all three subunits have very similar effects on function.

Mechanisms of proton translocation by Complex I have been outlined by several groups in recent years. The recent crystal structures of Complex I by the Sazanov group have motivated several proposals [41], including that the C-terminal helix of subunit L acts as a piston to drive conformational changes in the L, M and N subunits in response to redox reactions [17]. The conformational changes would then lead to proton translocation. A recent proposal by the Ohnishi group [42], [43] incorporates the possibility of such conformational changes, but emphasizes the redox reactions of two ubiquinones within Complex I to translocate two of the four protons per NADH by a direct proton pump mechanism. They proposed that one quinone, Q_Nf_, accepts 2 electrons from the N2 Fe-S center and 2 vectorial protons from the cytoplasmic surface (or mitochondrial matrix). It then passes 2 electrons to the second quinone and the 2 vectorial protons are released on the opposite side of the membrane in a “direct” proton pumping process. The second quinone, Q_Ns_, then accepts 2 scalar protons from the cytoplasmic surface to become ubiquinol, and enters the Q-pool.

**Figure 7:**
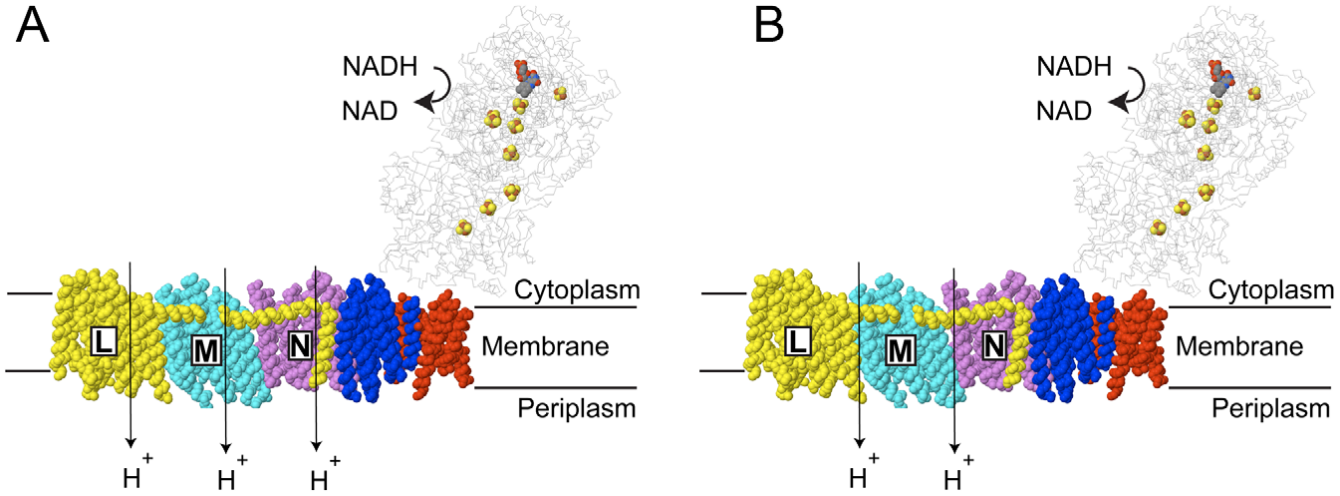
Two schematic views of the indirect proton translocation by Complex I. The peripheral arm is shown in thin wireframe with the flavin (FMN) and Fe-S centers in colored, space filling. The membrane subunits are shown in color: H (red), N (violet), M (cyan), L (yellow), and subunits A, J, and K are shown in blue. The protein structure is from the pdb file 3m9s for Complex I from *Thermus thermophilus* [17]. The C-terminal region of subunit L can be seen to contain a lateral helix that interacts with subunits M and N, and a final transmembrane helix that interacts with N at the junction with A, J or K subunits. (**A**) The L, M and N subunits are each suggested to translocate one proton per NADH. (**B**) An alternative view is that proton translocation occurs through the interaction of two subunits, resulting in a ratio of only 2 protons per NADH. The movement of protons would be facilitated by the conserved glutamic acids and lysines shown in Figure 1. The proton pathways would not necessarily occur along the interfaces.

The results presented here support the view that the L, M and N subunits have a common role in function. That might suggest that each operates independently to translocate one proton per enzyme cycle (NADH oxidation), as illustrated in Figure 7A. However, other observations allow consideration of a slightly different model. First is that mutagenesis of the highly conserved E133 in subunit N [31] did not affect enzyme function as did comparable substitutions of E144 in subunits L [35] and M [33], [34]. Since this residue is a prime candidate for proton translocation, it is possible that subunit N is not fully functional in that process. Second, in a recent analysis of subunit N (ND2) sequences, it was found that many metazoans are lacking three N-terminal transmembrane helices, and that a few are lacking the key glutamic acid (E133 in *E. coli*). Finally, in the *mrp* family of seven subunit antiporters, there are two subunits that are homologous to the L, M, and N subunits. Therefore, it seems possible that two subunits work together for ion translocation. Extending this view to Complex I, the steps of proton translocation might be carried out by the interactions of L:M and M:N. So, an alternative model is that the L, M, and N subunits are tightly coupled, and undergo similar conformational changes. In this model, they would work cooperatively to translocate 2 protons per NADH. This is illustrated in Figure 7B. Further work, including a higher resolution structure of Complex I, will be required to reveal the role of the long helical segment of subunit L, and the relative importance of subunit interactions in proton translocation.

## Materials and Methods

### Materials

Deamino-NADH, capsaicin, and decylubiquinone were from Sigma-Aldrich (St. Louis MO). DNA miniprep columns were from Qiagen (Carlsbad CA). PVDF (polyvinylidene difluoride) membranes, NBT (*p*-nitro blue tetrazolium chloride), BCIP (5-bromo-4-chloro-indolphosphate *p*-toluidine salt), SDS-polyacrylamide gels, low range molecular weight standards and the DC protein assay kit were from Bio-Rad (Hercules CA). ACMA was obtained from Invitrogen (Carlsbad CA). Rat (high affinity) anti-HA (hemagglutinin of influenza) monoclonal antibodies were from Roche (Indianapolis IN). Custom polyclonal antibodies against subunits L and M were prepared by Affinity BioReagents (Golden CO). These antibodies were raised in rabbits against peptide QTYSQPLWTWMSVGD (corresponding to residues 58–72) in subunit L and peptide GKAKSQIASQELPGM (corresponding to residues 446–460) in subunit M. Enzymes for molecular cloning were from New England Biolabs (Beverly MA). Oligonucleotides for mutagenesis and sequencing were synthesized by Operon Technologies, Inc (Huntsville AL). Sequencing of DNA was performed by Lone Star Labs (Houston TX). The QuikChange mutagenesis kit was from Stratagene (La Jolla CA).

### Plasmids, mutagenesis, growth and expression

Plasmids pLMN (8.10 kb) and pL'MN (6.57 kb) were used for construction of mutants. Both plasmids are derivatives of pUC19 (Amp^R^). They differ only in that pLMN contains full length genes of all three subunits (L, M, and N) where as pL'MN contains a 3′ truncated gene for L and full length genes for subunits M and N. pL'MN was constructed by isolating the 3.73 kb Pst I-Bgl II fragment from pAJW104 [44] and ligating to pUC19 digested with Pst I and BamH I at the polylinker region. pLMN was constructed from pL'MN by first introducing a unique BsrG I site between HinD III and Pst I using a linker (AGCTttgtacagactgacTGCA, where the lower case letters represent double-stranded nucleotides). Next, a 1.54 kb BsrG I-Pst I fragment from the *nuoL* region of pBA400 was isolated and ligated to pL'MN(BsrGI), previously digested with BsrG I and Pst I. Mutations in the L subunit were constructed in pLMN, and mutations in the M or N subunits were constructed in pL'MN. Plasmid pBA400 (derived from pACYC184, Cm^R^) was used as the wild type plasmid and it contains a full size *nuo* operon [45]. Mutations from the smaller plasmids (pLMN or pL'MN) were subcloned into the *nuo* operon of pBA400 for analysis of function, using Pst I and Asc I (2.81 kb fragment) for mutations in M or N, and BsrG I and Pst I (1.54 kb fragment) for mutations in L. For characterization of mutants pBA400 (mutant or wild type) was transformed into strain BA14 (*bglR*, *thi-1*, *rel-1*, Hfr Po1, Δ*nuoA-N*), a strain that lacks all subunits of Complex I [45]. For subcloning and mutagenesis, XL1-Blue (*recA*, *endA1*, *gyrA96*, *thi-1*, *hsdR17*, *supE44*, *relA1*, *lac* {F′ *proAB*, *lacI^q^* ZΔM15 Tn10 (*Tet^R^*)}) was used. Cultures were grown at 37°C in LB (1% tryptone, 0.5% yeast extract, and 0.5% NaCl) or 30°C in rich media (3% tryptone, 1.5% yeast extract, 0.15% NaCl and 1% (v/v) glycerol). Ampicillin (100 mg/l), tetracycline (12.5 mg/l), or chloramphenicol (40 mg/l) was added to the media as appropriate. Growth on M63 minimal salt media was supplemented with a single carbon source (acetate), to compare the growth of mutants reletive to the wild type. Acetate plates contained 1.36% KH_2_PO_4_, 0.2% (NH_4_)_2_SO_4_, 0.05% FeSO_4_•7H_2_O, 1.5% Agar, 0.02% MgSO_4_, 0.001% vitamin B1, and 0.2% potassium acetate. Growth was observed by visual inspection after 48 hours at 37°C.

### Preparation of Membrane Vesicles and Enzyme Assays

Membrane vesicles were prepared from cultures of all mutants and wild type grown in rich media at 30°C. Cultures were shaken at ∼230 rpm and harvested at A_600_ = 1.4. The cells were harvested, as previously described [32], [45]. For proton translocation assays membranes underwent a third centrifugation, 1 hour at 355,000×g. The membranes were tested for deamino-NADH driven proton translocation by measuring the fluorescence quenching of ACMA over the course of several minutes, using excitation and emission wavelengths of 410 and 490 nm respectively. Deamino-NADH oxidase activity assays and proton translocation assays were performed after the second centrifugation in 50 mM MOPS, 10 mM MgCl_2_, pH 7.3 at room temperature, using 150 µg/ml membrane protein. Deamino-NADH oxidase activity was assayed using oxygen as a terminal electron acceptor. The oxidase assays were started with 0.25 mM deamino-NADH (extinction coefficient 6.22 mM^−1^ cm^−1^) and the absorbance monitored at 340 nm for 2 minutes. Decylubiquinone was added from an ethanol stock to the reaction cuvette containing membranes, and the samples were incubated for several minutes at room temperature before addition of deamino-NADH. Complex I inhibitor capsaicin was added at 0.3 mM from a 100 mM ethanol stock. The uncoupler FCCP was added to a final concentration of 1 µM from a 1 mM ethanol stock. For proton translocation assays, ACMA was added to 1 µM, while other concentrations were the same as for oxidase assays. Ferricyanide reductase assays were conducted at room temperature and the absorbance monitored at 410 nm for 2 minutes in buffer containing 10 mM potassium phosphate (pH 7.0), 1 mM EDTA, 1 mM K_3_FeCN_6_, and 10 mM KCN [46]. 40–100 µg/ml of membrane protein was used in each assay. Ferricyanide was used as the terminal electron acceptor (extinction coefficient of 1.0 mM^−1^ cm^−1^). The assays were started with 0.15 mM deamino-NADH.

### Immunoblotting of mutants

40 µg of membrane fractions were incubated in 2% SDS (v/v), and 8 µl of loading dye (60 mM Tris HCl (pH 6.8), 25% glycerol, 14.4 mM 2-mercaptoethanol, 0.1% bromophenol blue) for 15 minutes. These were then subjected to SDS-PAGE for 1.25 hours at 150 V using 12% acrylamide gels and transferred to PVDF (polyvinylidene difluoride) membrane using a Trans-Blot apparatus (Bio-Rad) for 1 h at 100 V. The PVDF membrane was blocked with 5% powered milk in TBS/Tween 20 (0.05% Tween 20) for 1 h and then washed with TBS/Tween 20 three times. For subunit L, M, or N detection, the blocked PVDF membrane was incubated at room temperature for 2 hours with the rabbit custom antibodies diluted 1∶5000 for L, 1∶10,000 for M or with rat anti-HA serum, diluted 1∶5000 for subunit N detection. After washing three times with TBS/Tween 20, the blot was incubated with goat anti rabbit or anti-rat IgG-alkaline phosphatase conjugate at a dilution of 1∶1000 for 1 hour. After three more washings with TBS/Tween 20, color is developed with BCIP (5-Bromo- 4-chloro-3-indolyl phosphate) and NBT (nitro-blue tetrazolium) according to the manufacturer's instructions.
